# AT-RvD1 Promotes Resolution of Inflammation in NOD/ShiLtJ mice

**DOI:** 10.1038/srep45525

**Published:** 2017-03-31

**Authors:** Ching-Shuen Wang, Christina L. Maruyama, Justin T. Easley, Bryan G. Trump, Olga J. Baker

**Affiliations:** 1 School of Dentistry, University of Utah, Salt Lake City, UT, USA

## Abstract

Sjögren’s syndrome (SS) is a chronic inflammatory autoimmune disease characterized by diminished secretory function of the exocrine glands. Treatments for hyposalivation are limited to the use of saliva substitutes and medications that provide only temporary relief. In light of the high degree of need and the limitations of current therapies, development of alternative treatments to restore functioning is essential. Resolvins (Rv), which are highly potent lipid mediators, offer a viable alternative for better treating inflammatory diseases such as SS. The goal of this study was to determine whether systemic preventive treatment with Aspirin-triggered RvD1 (AT-RvD1) reduces inflammation and preserves secretory functioning in NOD/ShiLtJ SS-like mice. Our results indicate that systemic treatment with AT-RvD1 diminishes the progression of the disease in salivary epithelium from female mice as follows: (a) improves secretory function, (b) reduces pro-inflammatory molecule gene expression, (c) increases anti-inflammatory molecule gene expression and (d) induces M2 macrophage polarization. Finally, AT-RvD1 decreases lymphocytic infiltration into the salivary glands when used with small doses of the steroid, dexamethasone, and promotes the tissue healing process.

Sjögren’s syndrome (SS) is an autoimmune disease affecting approximately 1% of the general population and up to 3% of people over the age of fifty^1^, with women accounting for more than 90% of diagnosed cases. Although extensive investigation has been done to understand SS^2^, the causes of the disease are yet unknown and treatments remain largely ineffective. There are several hypotheses suggesting that genetic variants or environmental components may lead to SS, though the pathogenesis is likely due to a combination of many factors^3^. So far, the well-defined condition among all patients is chronic inflammation of the exocrine tissues. Despite not knowing the exact cause(s) of SS, resolution of the inflammation and/or alleviation of disease progression is the ultimate goal for current researchers. Resolution of inflammation is a tightly regulated process in the body with specific checkpoints that must be met for proper resolution^4^. Current therapies, such as anti-inflammatory drugs (*e.g*. steroids) showed promising results by suppressing inflammatory responses, but consequently pre-terminates pro-inflammatory responses that are essential for tissue resolution^5^,^6^. Therefore, it is unlikely that treatment with only anti-inflammatory drugs would achieve the ultimate goal of a “cure”; however, combinations with other potent therapies could control inflammation by targeting endogenous pathways. An alternative approach using endogenous lipid mediators (*e.g*. resolvins) to regulate inflammation has demonstrated significantly effective responses in treating many inflammatory related diseases^7^.

Resolvins (Rvs), which are highly potent lipid mediators, offer a viable alternative for better treating SS. Specifically, they limit inflammation by inhibiting key immunological processes in response to infection, injury and/or environmental challenges^8^ while preserving tissue integrity and promoting tissue repair^9^,^10^. Rvs and their precursors are potential drug candidates for treating a broad range of acute and chronic diseases caused by a failure to resolve inflammation^11^. Target conditions include autoimmune diseases^12^,^13^, allergic diseases^14^, degenerative inflammatory diseases (*e.g*., atherosclerosis)^15^, degenerative retinal diseases^16^ and chronic dry eye^17^. Naturally occurring Rv subtypes include the E series (derived from eicosapentaenoic acid or EPA), the D series (RvD1 and RvD2, both of which are derived from docosahexanoic acid or DHA) and six aspirin-triggered analogs of the D series (AT-RvD1-6), which are comparable in their properties to naturally occurring Rvs^18^. Our previous results narrowed the range of Rvs to a single candidate, RvD1, based on its therapeutic properties. Specifically, RvD1 biosynthetic pathways are expressed in both mouse and human salivary glands^19^. Furthermore, RvD1-mediated receptor-signaling molecules are also expressed and active in salivary epithelium^20^,^21^. Additionally, systemic treatment in the NOD/ShiLtJ SS-like mice with either AT-RvD1 or dexamethasone (DEX) caused down-regulation of SS-associated inflammatory genes and reduction of apoptosis^22^. Finally, it has been demonstrated that the 17R epimer of Resolvin D1 is longer acting *in vivo* because it is less susceptible to rapid inactivation by the eicosanoid oxidoreductase (PGDH). Together, these results led us to hypothesize that AT-RvD1-mediated signaling pathways may promote resolution of inflammation in SS-like mice^23^. Moreover, we hypothesized that a combined treatment of RvD1 and steroids (*e.g*. DEX) would achieve better outcomes by eliciting different anti-inflammatory pathways. Our results indicate that systemic treatment with AT-RvD1 limits the progression of the disease in salivary epithelium of female mice as evidenced by improved secretory function, reduced pro-inflammatory molecules expression, increased anti-inflammatory molecules expression and induced M2 macrophage polarization. Furthermore, these responses are enhanced when we use combined treatments of AT-RvD1 and DEX.

## Materials and Methods

### Experimental animals

Female and male NOD/ShiLtJ mice were treated twice a wk starting at 4 wk for 16 wk via tail vein injections with a vehicle control (EtOH, 8.9%) and AT-RvD1 (Cayman Chemical, Ann Arbor, MI) at 0.1 mg/kg. We used DEX (Sigma Aldrich, St. Louis, MO) at 8.25 mg/kg as a positive control and for a combination of AT-RvD1 and DEX (4.25 mg/kg). The doses of AT-RvD1 and EDX used in this study were chosen based on previous studies indicating that 0.1 mg/kg produces a significant downregulation of systemic inflammatory genes as compared to 0.05 mg/kg (data not shown). At 12 wk and 20 wk of age, the mice were anesthetized with 80–100 mg/kg Ketamine +5 mg/kg Xylazine and subsequently euthanized by abdominal exsanguination. 20 wk old untreated C57BL/6, NOD/ShiLtJ and *ALX*/*FPR2*^−/−^ mice were used for [Ca^2+^]_i_ measurements. The animal protocol (14-006007) was reviewed and approved by the Institutional Animal Care and Use Committee (IACUC) at University of Utah, according to the animal welfare act of the United States (7 U.S.C. 2131 et. seq.). All mouse experiments were carried out at the animal care facility of the University of Utah, Colorow 383 building, in accordance with approved guidelines.

### Measurement of stimulated salivary flow rate

Mice were anesthetized and injected with pilocarpine-HCl/PBS and isoproterenol (Sigma Aldrich) at 10 mg/kg via *i.p* to stimulate saliva secretion. Then, saliva was collected for 5 min using a 200 μl pipette and placed in a tube immediately on ice in the presence of a protease inhibitor cocktail (Sigma Aldrich). Both total weight and volume of saliva were measured by microbalance (Mettler Toledo, Columbus, OH) and pipette (Eppendorf AG, Hauppauge, NY), respectively. Statistical results were analyzed and plotted using Prism (GraphPad Software Inc., La Jolla, CA).

### Intracellular free calcium concentration [Ca^2+^]_i_ measurements

[Ca^2+^]_i_ measurements were taken to assess ALX/FPR2 receptor activity following stimulation with the following agonists: Carbachol (Cch; 100 μM), AT-RvD1 (100 ng/mL), as well as PBS (negative control). First, submandibular glands (SMGs) from C57BL/6, NOD/ShiLtJ, and ALX/FPR2 knockout mice were isolated and dissociated using a GentleMACS Tissue Dissociator (Miltenyi Biotec Inc., Sand Diego, CA), then plated on Cell-Tak (BD Biosciences, San Jose, CA) in 8-well chambers mounted on German borosilicate coverglasses (Nalge Nunc International, Penfield, NY). Cells were allowed to adhere to Cell-Tak for 30 mins, then were pre-loaded with fluo 2-AM (Invitrogen, Carlsbad, CA) for 20 min at 37 °C. Cells were washed three times with DMEM/F-12. [Ca^2+^]_i_ release was measured using a fluorescence microscope (Leica Microsystems) and time-lapse settings. Cch, AT-RvD1, and PBS were added to wells and cells were observed for changes in fluorescent intensity. Graphs plotting mean fluorescent intensity versus time were created using LAS (Leica Microsystems), Excel, and Graphpad Prism software.

### Immunofluorescence

A detailed procedure of deparafinization and antigen retrieval methods of 5 μm thick paraffin embedded mouse SMG sections can be obtained from a previous study^24^. SMG sections from different treatment groups were then blocked in 5% goat serum in PBS for 1 h at RT, and incubated at 4 °C with the following antibodies at 1:250 dilutions: anti-rabbit AQP5 (Abcam, Cambridge, MA) and anti-rabbit ZO-1 (Invitrogen) in 5% goat serum overnight. Then, they were incubated for 1 h with anti-rabbit Alexa Fluor 568 secondary antibody at 1:500 dilutions in 5% goat serum at RT. Subsequently, tissue sections were counter-stained with TO-PRO-3 Iodide nuclear stain (Invitrogen) at RT for 15 min at 1:1000 dilutions. Finally, specimens were analyzed using a confocal Zeiss LSM 700 microscope (Carl Zeiss, Oberkochen, Germany) at 10× magnifications. A total depth of 5 μm was acquired for each sample, and a total projection was visualized in the xy planes.

### Histopathological evaluation

At 12 wk and 20 wk of age, NOD/ShiLtJ mice were sacrificed and SMG were removed, sectioned, and stained with hematoxylin & eosin (Supplementary Figs 4 and 5). Grading of SMG histological sections was performed as described by Chisholm & Mason^25^. Specifically, tissue sections were then stained with H&E and visualized on a Leica DMI6000B Inverted Microscope, with a grading scale of 0–4 was utilized to assess the degree of lymphocytic infiltration. Grades correspond to the number of foci (aggregate of 50 or more lymphocytes) per 4 mm^2^ as follows: 0 = no infiltrates, 1 = slight infiltrate, 2 = moderate infiltrate or <1 focus, 3 = 1 focus per 4 mm^2^ and 4 = >1 focus per 4 mm^2^. Each point in the graph represents an average of the grades taken at three levels from a single mouse SMG (*i.e*., top, middle, and bottom) with a grade 3 indicating severe lymphocytic infiltration in SMG.

### Gene expression

A detailed cDNA preparation can be obtained from a previous study^24^. Briefly, total cDNA from SMG was diluted at 1:50 ratios and used as templates for qPCR. The reactions were carried out by adding the following reagents: 2.5 μl of each primer (stock 10 μm, Supplementary Table 1), 5 μl of 1:50 cDNA dilutions and 10 μl of 2× SYBR Green master mixes (Bio-Rad, Hercules, CA). PCR experiments were performed on 96 well plates, relative fold changes of gene expression were normalized using β-actin, and results were analyzed using Prism software (GraphPad Software Inc.).

### Statistical analysis

Data presented are means ± S.D. of results from three or more determinations. Prism software (Graphpad Software, Inc.) was used to perform t-test and one-way ANOVA statistical analyses. *P*-values equal to or less than 0.05 represent significant differences between experimental groups.

## Results

### Treatment with AT-RvD1 before disease onset maintains healthy saliva flow rates in female NOD/ShiLtJ mice

**Figure 1:**
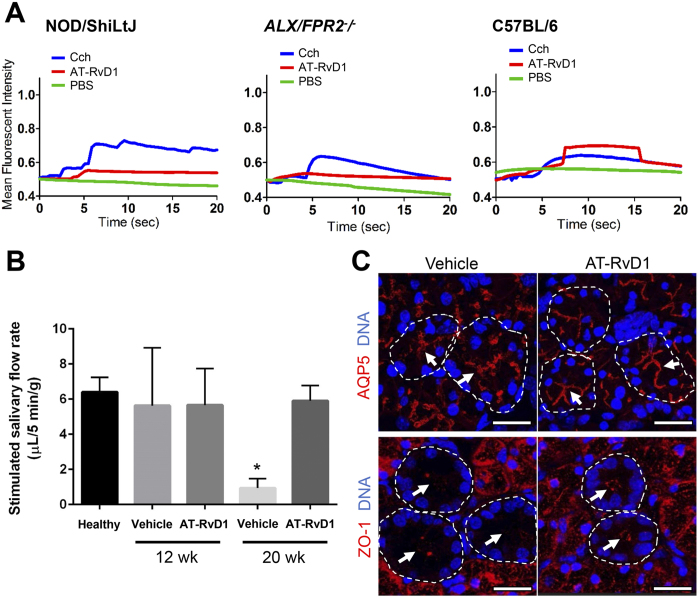
Treatment with AT-RvD1 prior to disease onset maintains healthy saliva flow rates and restores tissue integrity in female NOD/ShiLtJ mice. (**A**) Submandibular gland (SMG) cell clusters were obtained from 20 wk NOD/ShiLtJ, *ALX*/*FPR2*^−/−^ and C57BL/6 mice, mounted on coverglass and stimulated with AT-RvD1 (100 ng/mL), carbachol (Cch; positive control; 100 μM), or PBS (negative control) to measure changes in intracellular free calcium concentrations as described in the Material and Methods. Results shown are from a representative experiment from three or more determinations. (**B**) Salivary flow rates were calculated using female mice treated with either a vehicle control (8.9% EtOH in saline) or AT-RvD1 (0.1 mg/kg) at 4, 12 and 20 wk. Results from N = 3 mice were used per condition and data are expressed as mean ± SD, with *P < 0.05 indicating a significant difference from controls. (**C**) SMG from female NOD/ShiLtJ mice treated with AT-RvD1 or a vehicle control, were harvested, formalin-fixed, paraffin-embedded, and sectioned. Then, aquaporin 5 (AQP5) and zonula occludens-1 (ZO-1) were localized using confocal microscopy. TO-PRO-3 Iodide was used as a nucleic acid stain (blue). White arrows indicate luminal structures. Note that both AQP5 and ZO-1 appear disorganized in control mice. Representative fluorescent images from N = 3 are shown. Scale bars represent 50 µm.

In order to verify whether AT-RvD1 was able to stimulate its receptor, ALX/FPR2, in SMG from NOD/ShiLtJ mice, we measured [Ca^+2^]_i_ mobilization in response to this agonist in mouse SMG cells. As shown in Fig. 1A, SMG cells from 20 wk NOD/ShiLtJ mice were able to mobilize [Ca^2+^]_i_ in response to AT-RvD1. In contrast, SMG cells from 20 wk *ALX*/*FPR2*^−/−^ mice (used as a negative control) did not show such increase. However, SMG cells from both NOD/ShiltJ and *ALX*/*FPR2*^−/−^ mice responded to carbachol (Cch; acetylcholine analog; used as a positive control). Finally, SMG cells from C57BL/6 control mice showed responses to both AT-RvD1 and Cch (Fig. 1A and Supplementary Fig. 1A). In order to determine whether AT-RvD1 was able to preserve salivary secretory function in NOD/ShiLtJ mice, we treated them with AT-RvD1 before disease onset as described in the Materials and Methods, then measured saliva flow rate at 20 wk. As shown in Fig. 1B, female NOD/ShiLtJ mice treated with AT-RvD1 displayed saliva flow rates comparable to healthy controls (*i.e*., 4 wk old mice) and significantly higher flow rates when compared to vehicle controls. In contrast, male NOD/ShiLtJ mice subjected to the same treatment displayed saliva flow rates lower than healthy controls and comparable to vehicle controls (Supplementary Fig. 1b).

### Treatment with AT-RvD1 prior to disease onset maintains apical water channel protein, aquaporin-5 (AQP5), and tight junction zonula occludens-1 (ZO-1) localization

In order to determine whether saliva flow rates observed in female mice (Fig. 1B) were correlated with structural proteins related to secretion, we localized the water channel protein, aquaporin-5 (AQP5) and the tight junction protein, zonula occludens-1 (ZO-1) using confocal microscopy as described in Materials and Methods. As shown in Fig. 1C, female NOD/ShiltJ mice treated with a vehicle control displayed disorganized but apical staining of AQP5 (Fig. 1C, white arrows). Conversely, mice treated with AT-RvD1 showed organized apical staining of AQP5 (Fig. 1C, white arrows). Similarly, female NOD/ShiltJ mice treated with AT-RvD1 displayed organized apical staining of ZO-1 (Fig. 1C, white arrows) while vehicle-treated control mice displayed apical but disorganized staining of ZO-1 with low fluorescent intensity (Fig. 1C, white arrows).

### Treatment with AT-RvD1 prior to disease onset reduces lymphocytic infiltration in SMG

**Figure 2:**
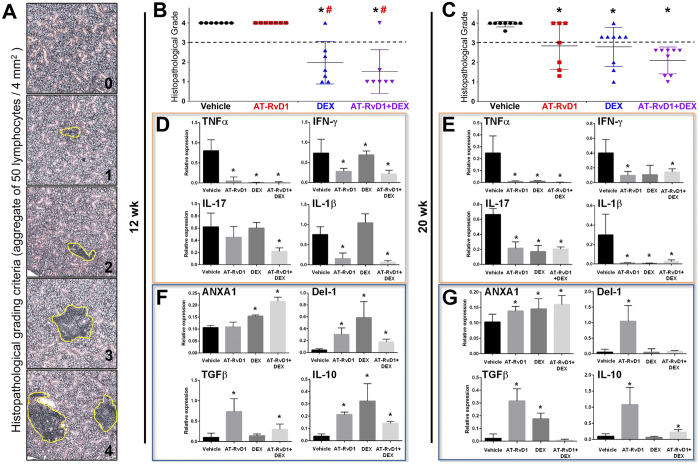
Treatment with AT-RvD1 prior to disease onset reduces lymphocytic infiltration and counter-regulates inflammatory responses in female NOD/ShiLtJ mice. Female NOD/ShiLtJ mice were treated with AT-RvD1 and a vehicle control with and without DEX as described in the Material and Methods. Submandibular glands (SMG) were then harvested and sectioned as described in the Material and Methods. Tissue sections were stained with H&E and visualized on a Leica DMI6000B Inverted Microscope. Sections were scored using a histopathological grading system as described in the Material and Methods. Each point in the graph represents an average of the grades taken at three levels from a single mouse SMG (*i.e*., top, middle, and bottom) with a dashed threshold line of grade 3 indicating severe lymphocytic infiltration in SMG. (**A**) Representative images showing a grading scale of 0–4 utilized to assess the degree of lymphocytic infiltration (highlighted in yellow circle). Groups are as follows: (**B**) Females at 12 wk, and (**C**) females at 20 wk. Results from N > 7 mice were used per condition and data are expressed as mean ± SD, with *P < 0.05 indicating a significant difference from vehicle, and ^#^*P* < 0.05 indicating a significant difference from RvD1. Additionally, RNA was isolated from the SMG for qPCR analysis of the following: (**D**) Pro-inflammatory cytokine profile at 12 wk, (**E**) pro-inflammatory cytokine profile at 20 wk, (**F**) anti-inflammatory molecule profile at 12 wk and (**G**) anti-inflammatory molecule profile at 20 wk. Results from N = 6 mice were used per condition and data are expressed as mean ± SD, with *P < 0.05 indicating a significant difference from controls. Orange boxes indicate pro-inflammatory cytokines and blue boxes indicate anti-inflammatory molecules.

A widely used histopathological grading system was used to score lymphocytic infiltration and representative images show varying degrees of inflammation in SMG (Fig. 2A). As shown in Fig. 2B and C, all female NOD/ShiLtJ mice treated with AT-RvD1 showed lymphocytic infiltration at 12 wk (similar to vehicle-treated control mice) with a slight reduction at 20 wk. Regarding male mice, no lymphocytic infiltration was observed at 12 wk, suggesting a slow disease onset (Supplementary Fig. 2A). However, 20 wk male NOD/ShiLtJ mice displayed lymphocytic infiltration (see vehicle-treated controls), and treatment with AT-RvD1 decreased lymphocytic infiltration in 7 out of 9 mice (Supplementary Fig. 2B).

Since our previous studies demonstrated that DEX significantly reduced lymphocytic infiltration in these mice, DEX was used as a positive control in this study. As shown in Fig. 2B, 12 wk female NOD/ShiLtJ mice treated with DEX showed a reduction in lymphocytic infiltration in 6 out of 7 mice. Furthermore, 20 wk female NOD/ShiLtJ mice treated with DEX showed a reduction in lymphocytic infiltration in 3 out of 8 mice (Fig. 2C). Regarding male mice, no lymphocytic infiltration was observed at 12 wk when treated with DEX, which was similar to the vehicle-treated controls, as expected (Supplementary Fig. 2A). Moreover, 20 wk male NOD/ShiLtJ mice treated with DEX also lacked observable lymphocytic infiltration (Supplementary Fig. 2B). Since we corroborated the positive effects of DEX in diminishing lymphocytic infiltration, we combined DEX with AT-RvD1. As shown in Fig. 2B, 12 wk female NOD/ShiLtJ mice treated with AT-RvD1 + DEX showed a reduction of lymphocytic infiltration in 5 out of 6 mice. Moreover, at 20 wk, female mice treated with AT-RvD1+DEX showed reduced lymphocytic infiltration in 9 out of 9 mice (Fig. 2C). Lastly, neither 12 wk nor 20 wk male NOD/ShiLtJ mice treated with AT-RvD1 + DEX showed lymphocytic infiltration (Supplementary Fig. 2A and B).

### Treatment with AT-RvD1 prior to disease onset reduces pro-inflammatory and increases anti-inflammatory molecule gene expression in SMG

Upregulation of pro-inflammatory cytokines has previously been observed in the NOD mouse^26^,^27^. To determine whether AT-RvD1 altered the inflammatory cytokine profiles, we performed a quantitative PCR analysis in SMG from female NOD/ShiLtJ mice. As shown in Fig. 2D, SMG from 12 wk female NOD/ShiLtJ mice treated with AT-RvD1 displayed significant downregulation of expression of the pro-inflammatory cytokines, TNFα, IL-1β and IFNγ, as compared to vehicle-treated controls. In addition, SMG from 20 wk female NOD/ShiLtJ mice treated with AT-RvD1 displayed a significant downregulation of the pro-inflammatory cytokines, TNFα, IL-1β, IFNγ and IL-17 as compared to vehicle-treated controls (Fig. 2E). In contrast, SMG from 20 wk male NOD/ShiLtJ mice treated with AT-RvD1 did not display these changes (Supplementary Fig. 2C).

Anti-inflammatory molecules were also investigated in this study. As shown in Fig. 2F, SMG from 12 wk female NOD/ShiLtJ mice treated with AT-RvD1 displayed a significant upregulation of Del-1 (endogenous leukocyte-endothelial adhesion inhibitor), TGFβ and IL-10 as compared to vehicle-treated controls. Moreover, SMG from 20 wk female NOD/ShiLtJ mice treated with AT-RvD1 displayed a significant upregulation of Annexin A1 (ANXA1), Del-1, TGFβ and IL-10 expression (Fig. 2G). However, SMG from 20 wk male NOD/ShiLtJ mice treated with AT-RvD1 did not show significant changes as compared to vehicle-treated controls (Supplementary Fig. 2D).

### Treatment with AT-RvD1 prior to disease onset triggers macrophage (Mϕ) M2 polarization in SMG

**Figure 3:**
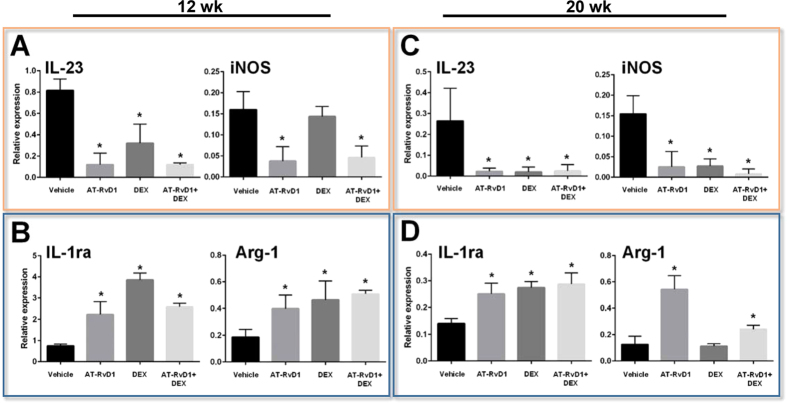
Treatment with AT-RvD1 prior to disease onset induces Mϕ polarization into pro-resolution M2-type in female NOD/ShiLtJ mice. Female NOD/ShiLtJ mice were treated with AT-RvD1 and a vehicle control with and without DEX as described in the Material and Methods. Submandibular glands (SMG) were frozen and gene expression was analyzed using qPCR. Gene expression profiles are as follows: (**A**) M1-type Mϕ from 12 wk female SMG, (**B**) M2-type Mϕ from 12 wk female SMG, (**C**) M1-type Mϕ from 20 wk female SMG, and (**D**) M2-type Mϕ from 20 wk female SMG. Orange boxes indicate genes associated with M1 Mϕ and blue boxes indicate genes associated with M2 Mϕ.

Previous studies have shown that treatment with AT-RvD1 polarizes pro-inflammatory M1-type macrophages (Mϕ) toward a pro-resolution M2-type in mouse and human cells^28^,^29^. Therefore, we aimed to characterize the Mϕ phenotype in SMG from different treatment groups. Specifically, gene signatures of either M1 (IL-23 and iNOS) or M2 (IL-1ra and Arg-1) type Mϕ were analyzed in this study. Our results showed that SMG from 12 wk female NOD/ShiLtJ mice treated with AT-RvD1 displayed a significant downregulation of M1-type Mϕ genes (IL-23 and iNOS, Fig. 3A) and upregulation of pro-resolution M2-type Mϕ genes (IL-1ra and Arg-1, Fig. 3B) as compared to vehicle-treated controls (Fig. 3A and B). Furthermore, SMG from 20 wk female NOD/ShiLtJ mice treated with AT-RvD1 demonstrated similar effects (*i.e*. M1-type downregulation/M2-type upregulation) as compared to vehicle-treated controls (Fig. 3C and D).

In contrast, SMG from 12 wk female NOD/ShiLtJ mice treated with DEX did not display downregulation of M1-type Mϕ genes (iNOS) but displayed upregulation of several anti-inflammatory genes (IL-1ra and Arg-1, Fig. 3A and B). SMG from 20 wk female NOD/ShiLtJ mice treated with DEX did show a significant downregulation of several M1-type Mϕ genes (IL-23 and iNOS) but only M2-type Mϕ genes were significantly upregulated (IL-1ra, Fig. 3C and D). Therefore, this specific gene signature of DEX treated mice does not demonstrate M2-type Mϕ polarization.

Similar to AT-RvD1 treatment, SMG from 12 wk female NOD/ShiLtJ mice treated with AT-RvD1 + DEX displayed a significant downregulation of M1-type Mϕ genes (IL-23 and iNOS) and a significant upregulation of M2-type Mϕ genes (IL-1ra and Arg-1, Fig. 3A and B) as compared to vehicle-treated controls. Furthermore, SMG from 20 wk female NOD/ShiLtJ mice treated with AT-RvD1 + DEX displayed gene signatures similar to 12 wk mice (Fig. 3C and D).

## Discussion

The present work demonstrates that ALX/FPR2 is functional in NOD/ShiLtJ mouse SMG as indicated by increases in [Ca^2+^]_i_ elicited by its ligand, AT-RvD1 (Fig. 1A). These results are consistent with previous studies showing that ALX/FPR2 is expressed and functional in the rat parotid Par-C10 cell line and in mouse SMG^20^,^21^. Note that in the innate immune system (*i.e*., human monocytes and neutrophils), ALX/FPR2-receptor agonists do not stimulate increases in intracellular calcium but instead activate MAPK signaling though phosphorylation^30^. In contrast, activation of the ALX/FPR2 with LXA_4_ and RvD1 stimulate intracellular calcium mobilization both in human and mouse goblet cells^31^. These results are important given that both salivary glands and conjunctival cells are controlled by acetylcholine^32^. Along with these results, AT-RvD1 treatment is likely to increase intracellular calcium concentration to maintain salivary flow rates in female but not male NOD/ShiLtJ mice. The lack of effect in male mice is attributable to a 2–4 week delay in symptom presentation as compared to females^33^.

The poorly organized AQP5 at the apical region observed in vehicle-treated control mice (Fig. 1C, white arrows) is consistent with previous studies indicating alteration of this protein in salivary glands from humans with SS and NOD mice^34^. In contrast, results showing apical staining of AQP5 in SMG from mice treated with AT-RvD1 (Fig. 1C, white arrows) indicates an organization pattern typical of a healthy acinus^35^. Regarding ZO-1, a significant reduction of ZO-1 fluorescent intensity in the apical region (Fig. 1C, white arrows) observed in vehicle-treated control mice is consistent with altered epithelial integrity associated with SS^36^. Conversely, the organized apical expression of this protein in SMG from mice treated with AT-RvD1 (Fig. 1C, white arrows), is typical of healthy salivary epithelium. Together, these results demonstrate that AT-RvD1 has a beneficial effect in preserving secretory proteins and thereby epithelial integrity in salivary glands.

Regarding chronic inflammation, previous studies demonstrated that female NOD/ShiLtJ mice display significant lymphocytic infiltration in SMG between 8 and 12 wk that persists for the remainder of the life span^37^. Along these lines, AT-RvD1 produced only a slight reduction of lymphocytic infiltration in 20 wk female mice and a marked reduction in male mice (Fig. 2C). The poor reduction of lymphocytic infiltration in female SMG may be due to the short half-life of AT-RvD1 in plasma given its rapid hydrolysis^18^. The significant reduction observed in male SMG may be due to the slower disease onset in terms of glandular inflammation (Supplementary Fig. 2A and B); thus it is possible that the same dose of AT-RvD1 works more effectively in balancing the inflammatory response in males. Interestingly, when combining AT-RvD1 with DEX, we observed a significant reduction of lymphocytic infiltration in both males and females. These results are likely due to an upregulation of Annexin A1 (ANXA1) caused by DEX. Particularly, radiolabeled competition assays demonstrated that ANXA1 directly interacts with ALX/FPR2^38^. Moreover, the new concept of combining resolvins with other drugs has been demonstrated to be highly effective in resolving inflammation^39^. Therefore, it is plausible that DEX has an additive effect together with AT-RvD1, thereby increasing ALX/FPR2 signaling and consequently reducing infiltration of inflammatory cells into the gland (Fig. 2F and G). However, future studies will aim to confirm this notion. Finally, it is important to mention that resolvins in general are more potent than DEX and other anti-inflammatory drugs *in vivo*^40^,^41^,^42^.

Recently our pilot study showed that treatment with AT-RvD1 reduces gene expression of several pro-inflammatory cytokines (*e.g*., TNFα, IL-2, -4, -5, and -12β) as well as apoptotic levels in SMG from NOD/ShiLtJ mice^43^. In the current study, we were able to show that treatment with AT-RvD1 not only reduced pro-inflammatory molecule gene expression levels (TNFα, IL-1β, IFN-γ and IL-17) but also enhanced anti-inflammatory molecule gene expression levels (ANXA1, Del-1, TGFβ and IL-10). While we do not yet fully understand this mechanism, it is tempting to speculate that AT-RvD1 downregulates IL-17 leading to upregulation of Del-1, similar to what occurs in human endothelial cells and in a mouse model of inflammatory periodontitis^44^,^45^.

It is well established that immunoregulation under physiological conditions produces moderate acute inflammation in response to injury or infection. Inflammation is then followed by pro-resolution mechanisms and tissue healing. For this to occur, macrophages must switch from a pro-inflammatory M1 class to the pro-resolving M2 class^46^,^47^. NOD/ShiLtJ mice exhibit dysregulated functioning of lymphocytes and macrophages^48^, and thus, impaired wound healing. Interestingly, we observed significant changes in gene regulation suggestive of macrophage class switching^28^,^29^ in NOD/ShiLtJ mice treated with AT-RvD1. Specifically, treatment with AT-RvD1 switched gene regulation profiles from pro-inflammatory M1 type Mϕ into pro-resolution M2 type Mϕ (Fig. 3); however, a reduction in lymphocytic infiltration was not observed during the early phases of the treatment (Fig. 2B). DEX-treated mice did not exhibit the same Mϕ class-switching (Fig. 3), despite promising reductions in lymphocytic infiltration (at 12 wk; Fig. 2B) and pro-inflammatory cytokine expression (at 20 wk; Fig. 2D). Moreover, DEX did not preserve salivary epithelial tissue integrity, as evidenced by disorganized AQP5 and ZO-1 localization (Supplementary Fig. 3). It is conceivable that treatment with DEX inhibits inflammatory responses that are critical for tissue healing. Remarkably, when a combination of AT-RvD1 and DEX was used, NOD/ShiLtJ mice exhibited improvements in both lymphocytic infiltration and epithelial integrity at both 12 wk and 20 wk (Fig. 2B and C, Supplementary Figs 3 and 4).

**Figure 4:**
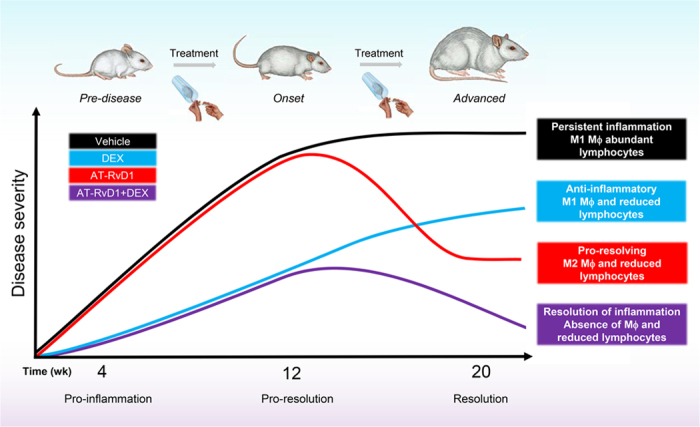
Effects of AT-RvD1 and DEX in mediating inflammatory responses and promoting resolution of inflammation in SMG. Without any therapeutic intervention, NOD/ShiLtJ mice develop severe SS-like symptoms (animal schematic at top). However, AT-RvD1 plays a substantial role alone and in combination with DEX in reducing inflammation, reducing lymphocytic infiltration, increasing anti-inflammatory responses and increasing pro-resolution M2-type Mϕ polarization in SS-like NOD mice. The schematic diagram was drawn by authors Olga J. Baker, Ching-Shuen Wang and Christina L. Maruyama.

In conclusion, resolution initiated via activation of the ALX/FPR2 with AT-RvD1 has a significant impact in controlling tissue homeostasis, thus preventing acute inflammation from progressing to chronic autoimmunity. Specifically, we demonstrated a critical role of AT-RvD1 in resolving inflammation associated with SS-like disease progression in the mouse SMG (Fig. 4). This finding is consistent with our previous study showing that without the protection of ALX/FPR2, persistent salivary gland epithelial inflammation is observed^24^. Therefore, intact ALX/FPR2 signaling is crucial to maintain salivary gland homeostasis. Additionally, tissue healing could be largely accelerated by treatments using AT-RvD1 together with low doses of DEX. In this study, we demonstrated that the autoimmune exocrinopathy observed in the NOD/ShiLtJ mice can be prevented by using AT-RvD1 and DEX. Finally, these studies provide a better understanding of RvD1 signaling pathways associated with SS. Future experiments using this treatment at disease onset are timely and warranted.

## Additional Information

**How to cite this article:** Wang, C.-S. *et al*. AT-RvD1 Promotes Resolution of Inflammation in NOD/ShiLtJ mice. *Sci. Rep.* **7**, 45525; doi: 10.1038/srep45525 (2017).

**Publisher's note:** Springer Nature remains neutral with regard to jurisdictional claims in published maps and institutional affiliations.

## Supplementary Material

Supplementary Information
