# Author Correction: AT-RvD1 Promotes Resolution of Inflammation in NOD/ShiLtJ mice

**DOI:** 10.1038/s41598-018-33005-w

**Published:** 2018-10-16

**Authors:** Ching-Shuen Wang, Christina L. Maruyama, Justin T. Easley, Bryan G. Trump, Olga J. Baker

**Affiliations:** School of Dentistry, University of Utah, Salt Lake City, UT USA

Correction to: *Scientific Reports* 10.1038/srep45525, published online 31 March 2017

This Article contains a typographical error in the Materials and Methods section under the subheading ‘Experimental animals’ where,

“The doses of AT-RvD1 and EDX used in this study…”

should read:

“The doses of AT-RvD1 and DEX used in this study…”

