# Supplementary material for: AT-RvD1 Promotes Resolution of Inflammation in NOD/ShiLtJ mice
Source: Sci Rep. 2017 Mar 31;7:45525. doi: 10.1038/srep45525 (PMC5374540; doi:10.1038/srep45525)
Supplement: Supplementary Information [file srep45525-s1.doc]

**Supplementary Information**

**AT-RvD1 Promotes Resolution of Inflammation in NOD/ShiLtJ mice**

Ching-Shuen Wang^1§^, Christina L. Maruyama^1§^, Justin T. Easley^1^, Bryan G. Trump^1^ and Olga J. Baker^1^*

§: Both authors contributed equally to this work.

^1^School of Dentistry, University of Utah, Salt Lake City, UT, USA

**Supplementary Table 1.**

**
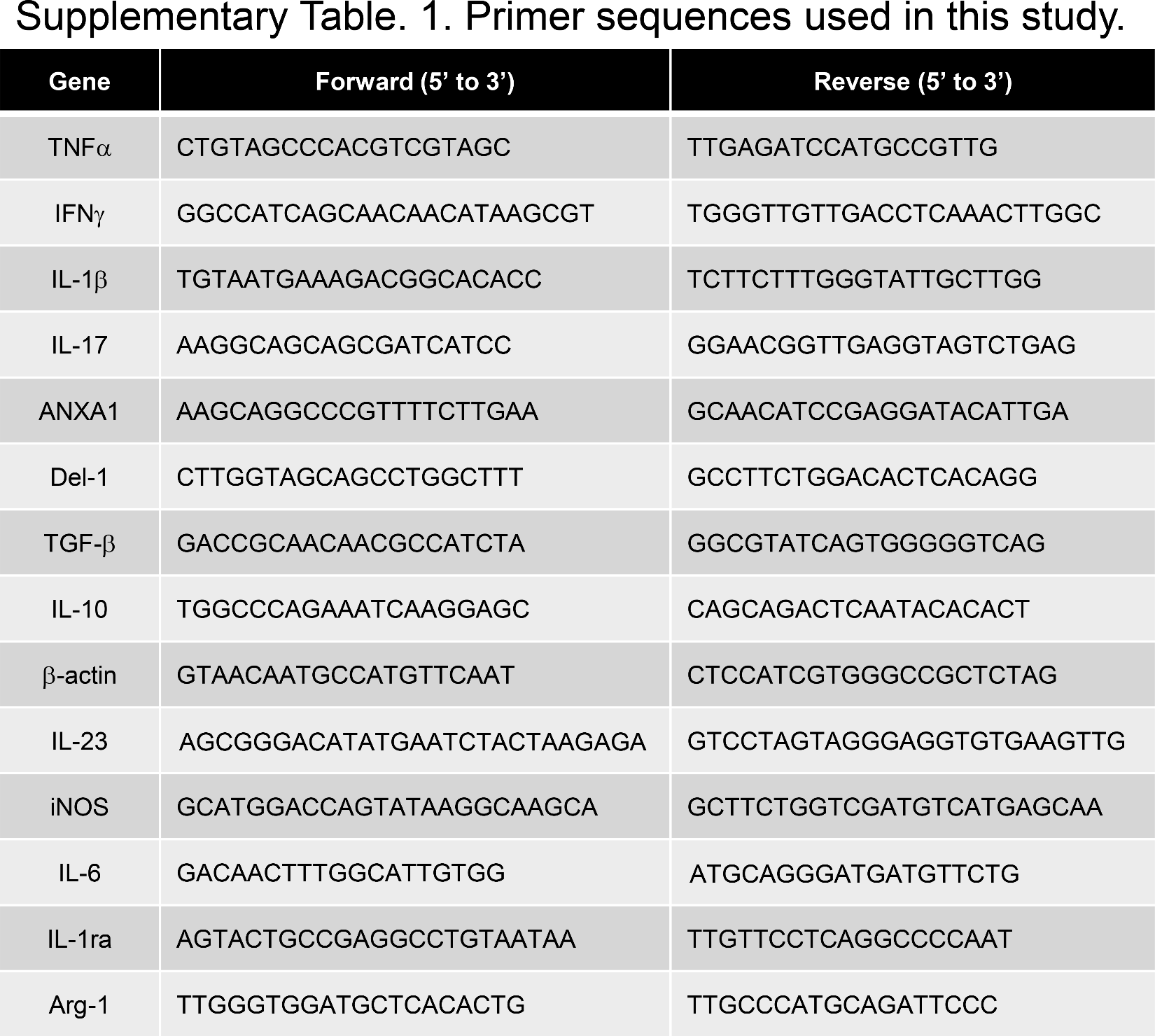
**

**
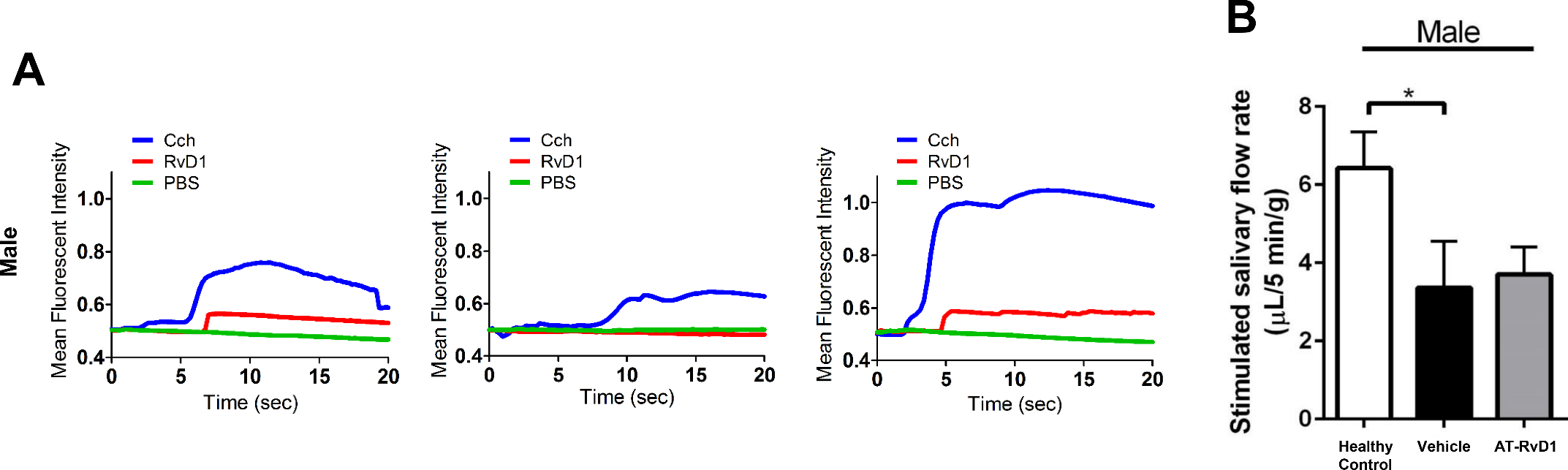
**

**Supplementary Fig. 1. Treatment with AT-RvD1 prior to disease onset (4 wk) did not affect saliva flow rates in male NOD/ShiLtJ mice. (A)** SMG cell clusters were obtained from 20 wk NOD/ShiLtJ, *ALX/FPR2^-/-^* and C57BL/6 mice, mounted on coverglass and stimulated with AT-RvD1, carbachol (Cch), or PBS to measure changes in intracellular free calcium concentrations described in the Materials and Methods. Results shown are from a representative experiment from three or more determinations. **(B)** Salivary flow rates were calculated using male mice treated with either a vehicle control or AT-RvD1 at 4 wk (healthy control) and 20 wk. Results from N=3 mice were used per condition and data are expressed as mean ± SD, with **P* < 0.05 and ****P* < 0.001 indicating a significant difference from controls.

**
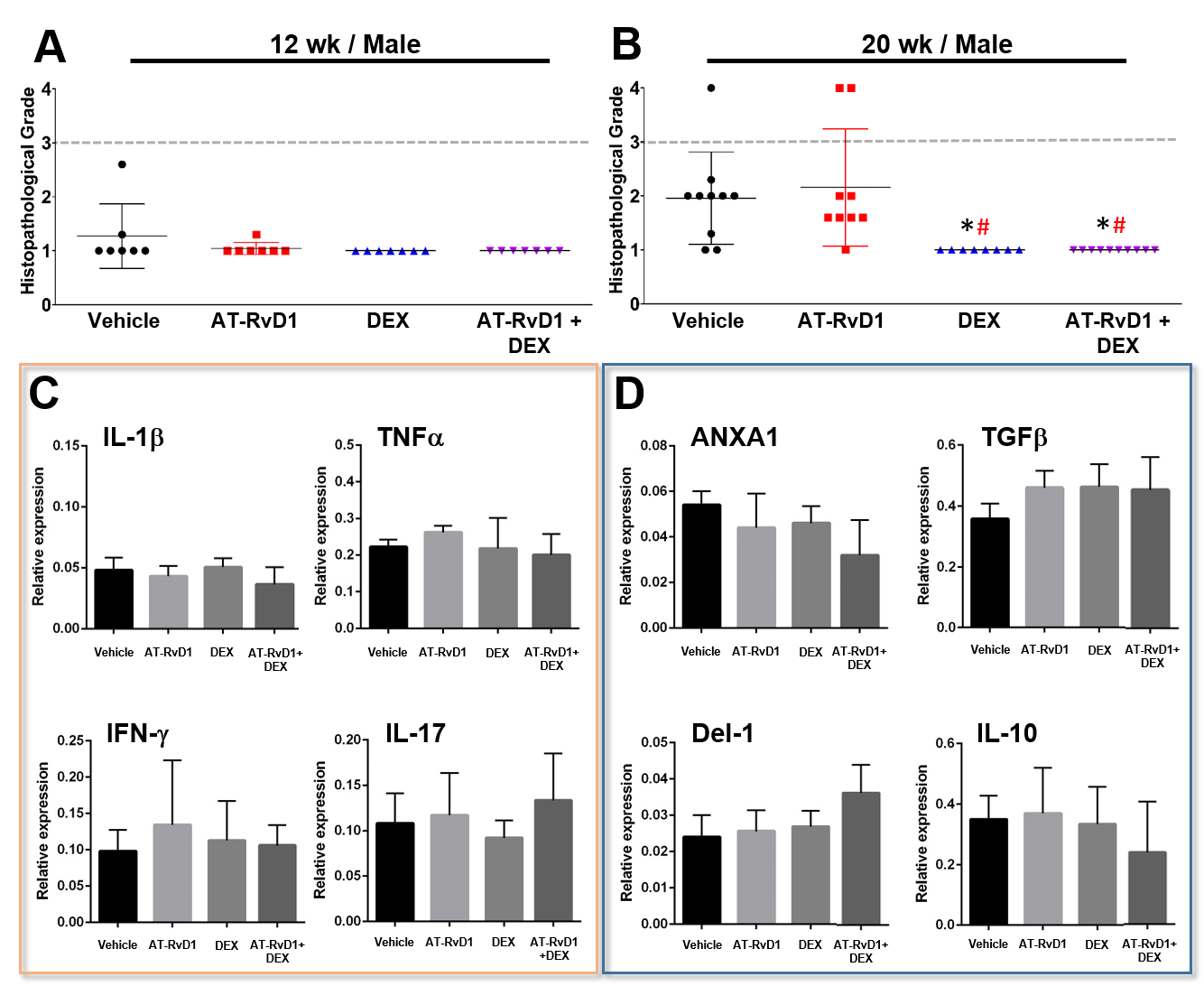
**

**Supplemental Fig. 2. Treatment with AT-RvD1 prior to disease onset reduces lymphocytic infiltration and counter-regulates inflammatory responses in male NOD/ShiLtJ mice.** Male NOD/ShiLtJ mice were treated with AT-RvD1 and a vehicle control with and without DEX as described in the Materials and Methods. SMGs were then harvested and sectioned as described in the Materials and Methods. Tissue sections were stained with H&E and visualized on a Leica DMI6000B Inverted Microscope. Sections were scored using a histopathological grading system as described in the Materials and Methods. Each point represents a composite analysis of a single SMG from a given mouse in which grades from three glandular regions (*i.e.,* top, middle, and bottom) were averaged. Groups are as follows: **(A)** males at 12 wk, and **(B)** males at 20 wk. A dashed threshold line of grade 3 indicates severe lymphocytic infiltration in SMG. Results from N>7 mice were used per condition and data are expressed as mean ± SD, with **P* < 0.05 indicating a significant difference from vehicle, and ^#^*P* < 0.05 indicating a significant difference from RvD1. Additionally, RNA was isolated from the SMG for qPCR analysis of the **(C)** pro-inflammatory cytokine profile at 20 wk and **(D)** anti-inflammatory cytokine profile at 20 wk. Results from N=6 mice were used per condition and data are expressed as mean ± SD, with **P* < 0.05 indicating a significant difference from controls.


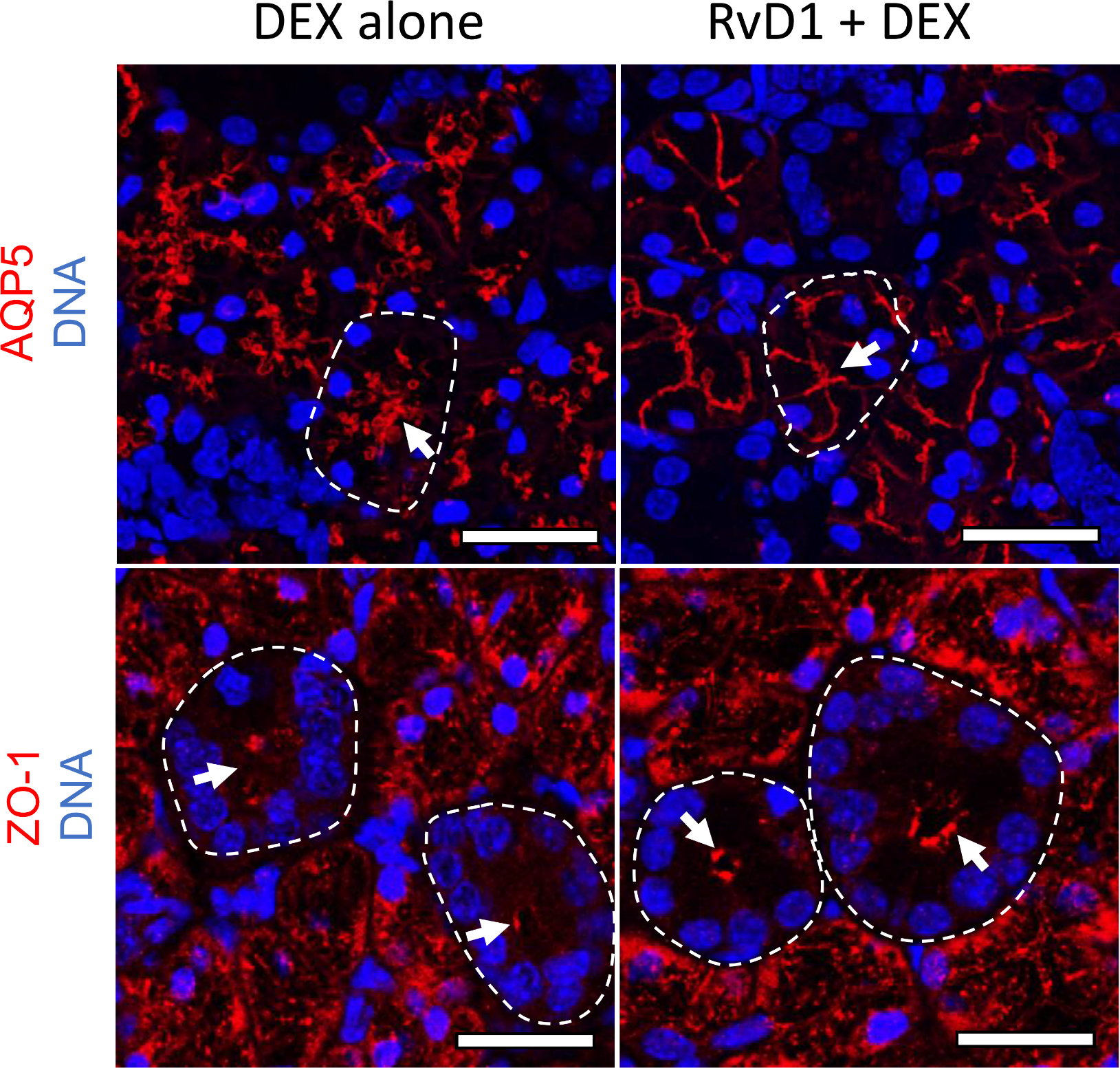


**Supplementary Fig. 3. Treatment with AT-RvD1 with DEX prior to disease onset (4 wk) restores tissue integrity in female NOD/ShiLtJ mice.** Submandibular glands from female NOD/ShiLtJ mice treated with AT-RvD1, DEX, or a combination of the two treatments were harvested, formalin-fixed, paraffin-embedded, and sectioned. Then, aquaporin 5 (AQP5) and zonula occludens-1 (ZO-1) were localized using confocal microscopy. TO-PRO-3 Iodide was used as a nucleic acid stain (blue). White arrows indicate luminal structures. Note that both AQP5 and ZO-1 appear disorganized in DEX-treated mice. Representative fluorescent images from N=3 are shown.

**
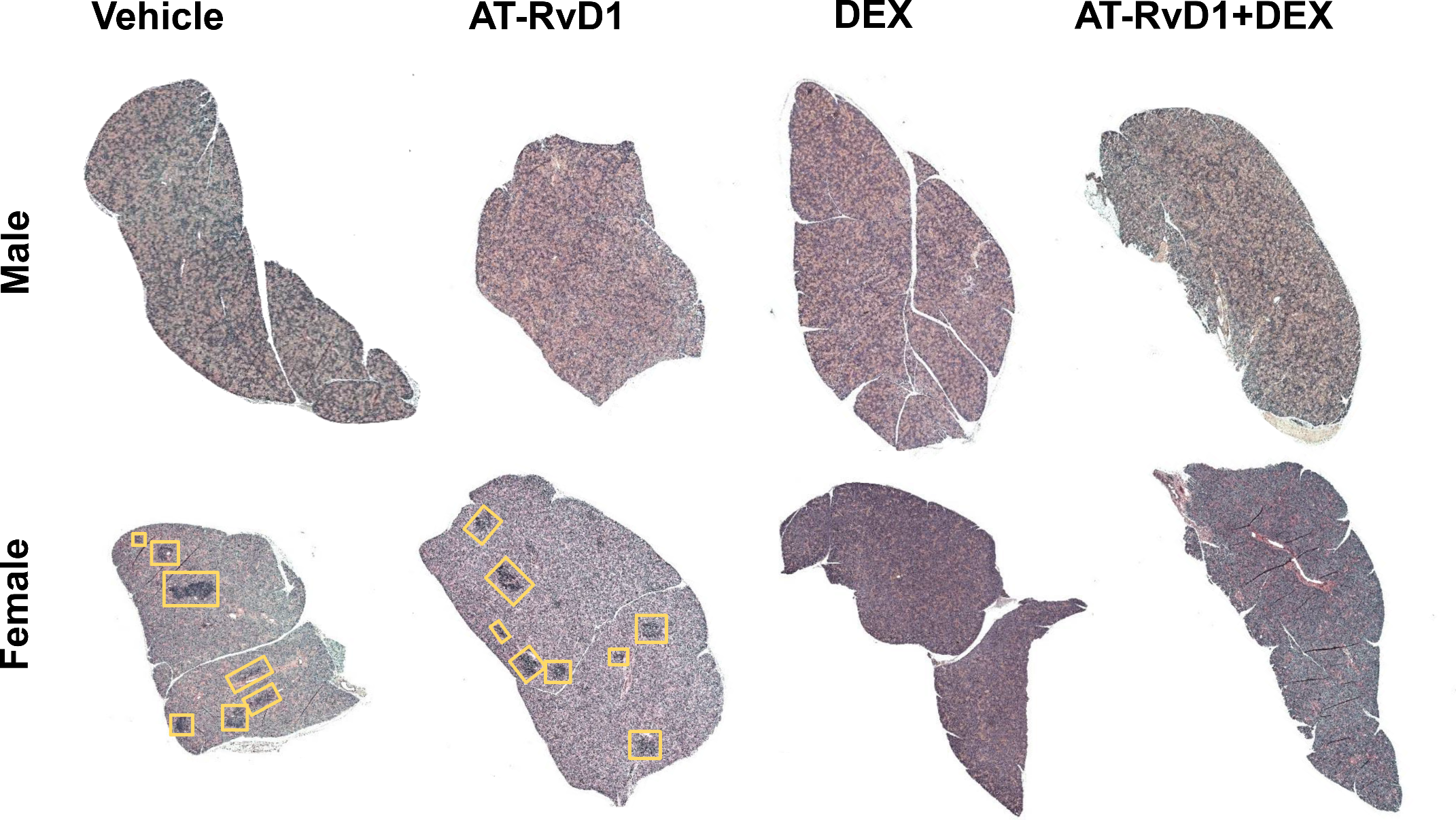
**

**Supplemental Fig. 4. Treatment with AT-RvD1 prior to disease onset reduces lymphocytic infiltration in both female and male NOD/ShiLtJ mice.** Animals were treated with AT-RvD1 and a vehicle control with and without DEX as described in the Materials and Methods. SMGs harvested from 12 wk old mice were then formalin-fixed, paraffin-embedded, and sectioned. Tissue sections were stained with H&E and visualized on a Leica DMI6000B Inverted Microscope. Tissue sections shown are representative of N=7 where yellow-boxes designate aggregates of lymphocytes.

**
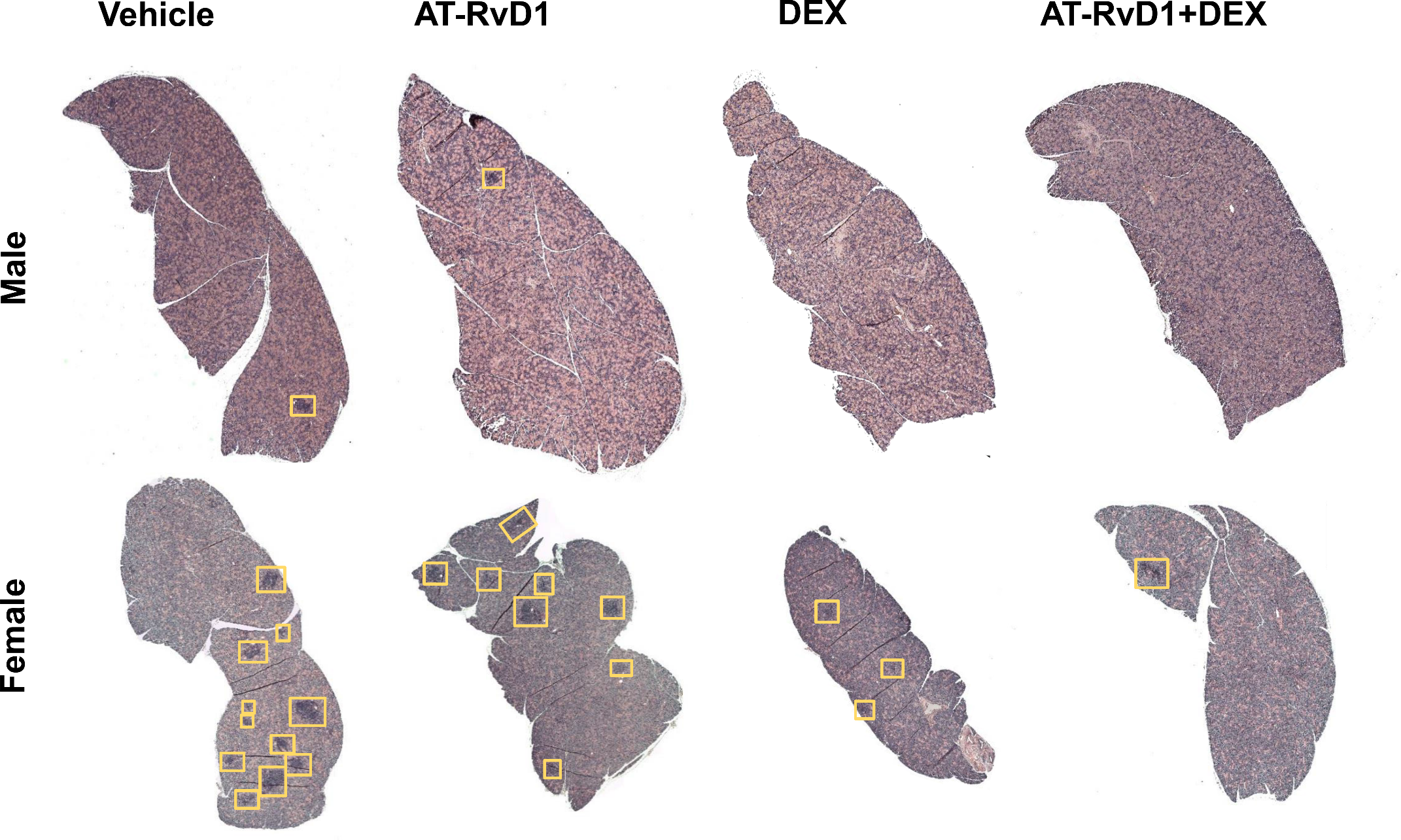
**

**Supplemental Fig. 5. Treatment with AT-RvD1 prior to disease onset reduces lymphocytic infiltration in both female and male NOD/ShiLtJ mice.** Animals were treated with AT-RvD1 and a vehicle control with and without DEX as described in the Materials and Methods. SMGs harvested from 20 wk old mice were then formalin-fixed, paraffin-embedded, and sectioned. Tissue sections were stained with H&E and visualized on a Leica DMI6000B Inverted Microscope. Tissue sections shown are representative of N=7 where yellow-boxes designate aggregates of lymphocytes.
